# Modelling the impact of climate and the environment on the spatiotemporal dynamics of Lyme borreliosis in Germany

**DOI:** 10.1016/j.ebiom.2025.105701

**Published:** 2025-04-28

**Authors:** Martín Lotto Batista, Bruno Carvalho, Rory Gibb, Balakrishnan Solaraju-Murali, Stefan Flasche, Stefanie Castell, Stéphane Ghozzi, Rachel Lowe

**Affiliations:** a Barcelona Supercomputing Center (BSC), Barcelona, Spain; b Department for Epidemiology, Helmholtz Centre for Infection Research, Brunswick, Germany; c Centre for Biodiversity and Environment Research, Department of Genetics, Evolution & Environment, University College London, London, United Kingdom; d Centre for Mathematical Modelling of Infectious Diseases, London School of Hygiene & Tropical Medicine, London, United Kingdom; e Centre for Global Health, Charite, Universitaetsmedizin Berlin, Berlin, Germany; f Centre on Climate Change and Planetary Health, London School of Hygiene & Tropical Medicine, London, United Kingdom; g Catalan Institution for Research and Advanced Studies (ICREA), Barcelona, Spain

**Keywords:** Lyme borreliosis, Climate change, Bayesian modelling, Tick-borne diseases, Germany

## Abstract

**Background:**

Lyme borreliosis (LB) is a predominant vector-borne disease in Europe, with Germany reporting endemic circulation for at least the past two decades. Climatic and environmental conditions are key drivers of tick activity, and human exposure to tick bites. Understanding the climatic and environmental factors driving LB dynamics can help devise decision-support tools to guide interventions and adaptation strategies.

**Methods:**

Using a Bayesian modelling framework, we assessed the delayed and nonlinear associations between climate variation and land use change and monthly LB case counts from the German national notification system at a district level from 2009 to 2022. We evaluated the predictive performance of our model and then predicted risk trends in states without mandatory notification. We then used the fitted risk function for maximum temperature to assess long-term trends in relative risk since the 1950s.

**Findings:**

Our analyses revealed that climate and environmental factors are positively associated with LB cases reported to the national notification system. Maximum temperature between 10.5 °C and 26.3 °C two to four months prior, relative humidity levels exceeding 78.8% six months prior, and exceptionally wet conditions accumulated over three months, lagged by one month, were associated with an increased risk of LB. The effect of relative humidity was only relevant in areas suitable for deer population, potentially linked to tick survival. Predictions from our model identified significant increasing trends in Schleswig–Holstein, Hamburg, and Lower Saxony, three states without mandatory case notification. We also observed an increasing trend in maximum-temperature related LB relative risk in all Federal States, with the largest percentage change in the period 2013–2022 in northern districts, compared to 1951–1970.

**Interpretation:**

Our study underscores the role of climatic variables as potential drivers of LB risk in Germany. We identified optimal conditions that may be related to human exposure and tick survival and detected long-term upward trends nationwide, including in areas without mandatory notification. This decision-support modelling framework emphasises the added value of expanding LB surveillance in Germany and across Europe to address the emerging risk of tick-borne infectious diseases.

**Funding:**

10.13039/501100009318Helmholtz Association, Helmholtz Climate Initiative, 10.13039/100010269Wellcome Trust, 10.13039/501100000288Royal Society, and 10.13039/100018693Horizon Europe.

**Research in context**

**Evidence before this study**

Approximately 200,000 people become infected with Lyme borreliosis (LB) every year in Western Europe, leading to long term complications that pose large financial costs to public health agencies. There is evidence of an expansion of the ticks that transmit LB towards higher latitudes and altitudes as temperature increases, resulting in earlier onset of the transmission season with a longer duration. By May 2024, the PubMed search string "(borreliosis) AND (climate) AND (model) AND (Europe)" provided 59 publications. After refining our search to include only publications in English assessing the influence of climate or the environment on LB, we extracted 33 publications spanning from 2006 to 2023. Among these, 26 focused on tick-related risk, while only 7 explored climate-related disease dynamics. The evidence suggests that LB and its vector are indeed influenced by climate processes, although uncertainties remain regarding exposure mechanisms and the optimal climatic and environmental conditions associated with disease transmission.

**Added value of this study**

This study quantifies the association between climate and environmental factors, and the dynamics of LB using national notification data from Germany. We identified climatic and environmental characteristics that are associated with elevated risk. Our findings indicate that maximum temperature between 10.5 °C and 26.3 °C, two to four months prior, relative humidity levels exceeding 78.8% six months prior, and exceptionally wet conditions one month prior, are optimal for increased LB risk. Our results suggest that relative humidity is related to higher LB risk in deer suitable areas, potentially linked to tick survival. Additionally, we observed an upward trend in the maximum temperature driven component of LB relative risk since 1951, across the country.

**Implications of all the available evidence**

The differential impact of climate conditions on LB, particularly in regions suitable and unsuitable for tick hosts, provides key information for planning of public health interventions, urban expansion, and biodiversity conservation. Our results highlight the benefit of developing tailored, region-specific public health protection strategies, taking into consideration landscapes suitable for deer and hence, tick populations. Moreover, our findings suggest a lengthening of the transmission season as temperatures become milder during winter and autumn. Lastly, our results underscore the importance of understanding how a changing climate affects disease risk and of monitoring shifts in the spatiotemporal dynamics of LB in Germany, Europe, and beyond.

## Introduction

Lyme borreliosis (LB) is one of the most prevalent vector-borne diseases in Europe, with approximately 200,000 cases reported each year in Western Europe alone.^1^ It is caused by bacteria of the complex *Borrelia burgdorferii* s.l., transmitted to humans through bites from infected hard ticks of the genus *Ixodes*.^2^ Although symptomatic infections are not common, the most frequent symptom is the Lyme rash, formally known as erythema migrans, a self-limited skin rash that appears in the first few days after infection.^3^ More disseminated manifestations may compromise the nervous, cardiac, or musculoskeletal systems, as well as the skin.^4^ Due to the multiple clinical presentations and potentially long-lasting symptoms, LB can lead to a considerable reduction in the quality of life when left untreated.^5^

**Fig. 1:**
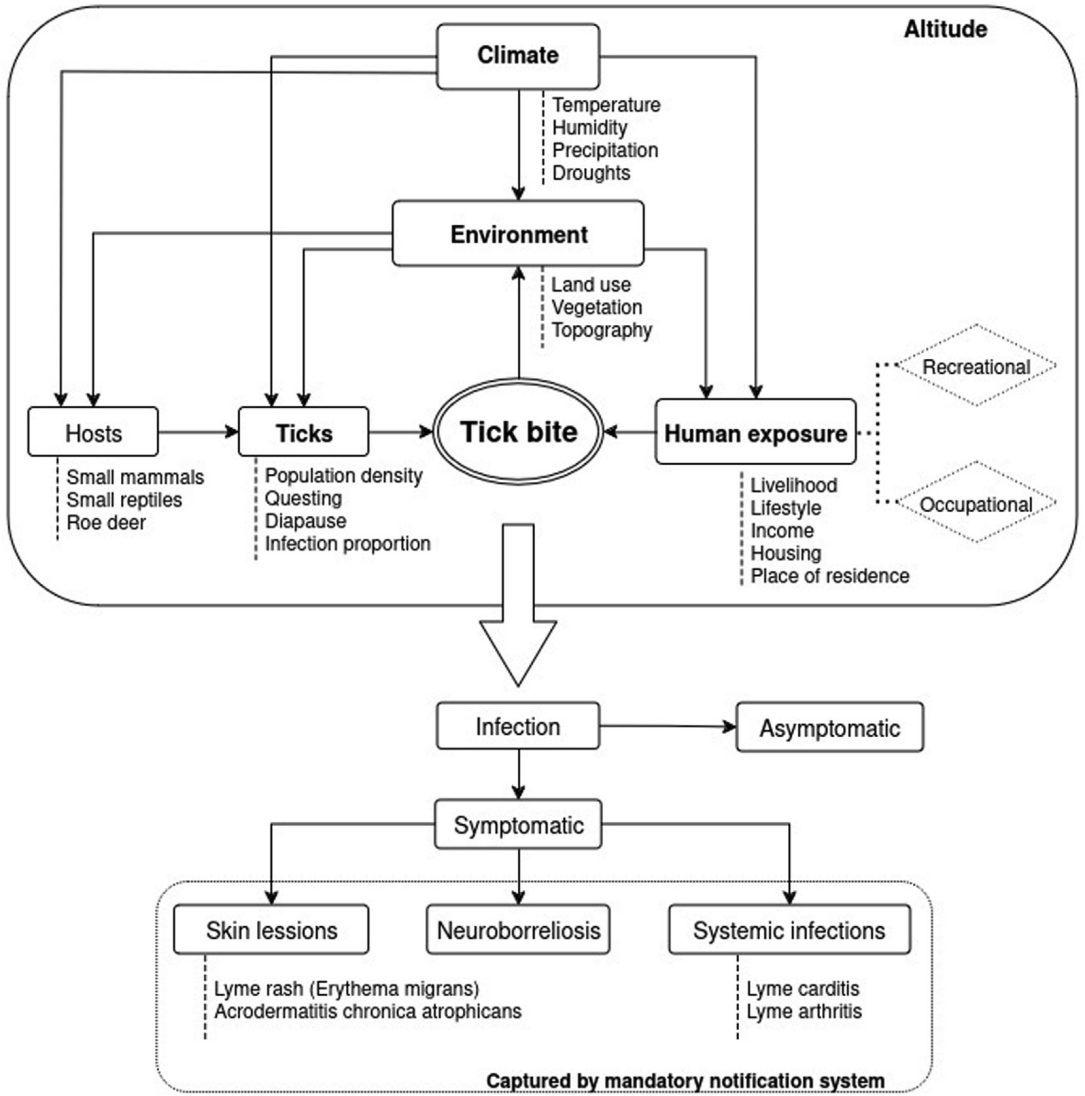
**Schematic representation of the transmission of Lyme borreliosis.** Abiotic processes, such as climate and the environment, regulate the activity and population size of ticks, and influence the amount of time humans spend in tick-suitable areas. Additionally, occupational, and recreational exposure are influenced by the level of income, as well as the type of jobs and activities people might engage in outdoors. In suitable conditions, questing ticks accidentally bite humans, which can lead to LB. More than 90% of the infections are asymptomatic. Symptomatic cases are often characterised by the presence of the Lyme rash or one of the less frequent presentations, such as neuroborreliosis, Lyme carditis, or Lyme arthritis, among others. Acute clinical manifestations such as Erythema migrans, acute neuroborreliosis and acute Lyme-arthritis are prone to be captured by the passive surveillance systems.

The main vector in Europe is the hard tick *Ixodes ricinus*.^3^ These ectotherm parasites have a life cycle with three developmental stages, from larva, nymph, to adult, involving a specific range of hosts.^6^ For instance, while larvae feed on small animals, such as squirrels and rodents, adults prefer large mammals, like deer.^6^ The feeding behaviour, also known as questing, involves ticks climbing up the low vegetation and attaching to a suitable host when they are nearby. Once ticks have attached to the host’s skin, they feed until becoming engorged, returning to the ground afterwards to initiate the transition to the next stage or to lay eggs.^6^ It is during questing that ticks can attach to humans and transmit pathogens (Fig. 1).

Climatic and environmental conditions are key drivers of tick activity.^7^ While waiting for a host, ticks are vulnerable to desiccation, hence requiring sufficiently humid conditions to survive.^8^ Ticks overcome desiccation by climbing down the vegetation to rehydrate in the detritus layer, as well as to avoid sun exposure.^6^ Also, ticks require at least 6 °C to start questing, extending up to 35 °C with sufficient humidity. As temperatures drop and days shorten, ticks enter the diapause phase to survive adverse winter conditions.^6^

There is evidence of an expansion of ticks towards higher latitudes and altitudes in Europe due to milder conditions in previously non-habitable areas.^9^ For instance, long-term field monitoring of ticks detected altitudinal shifts in a mountain range of the Czech Republic, where ticks carrying *B. burgdorferii* were found at 1065 m above sea level (m.a.s.l.), and in the northern Apennines of Italy, at 1650 m.a.s.l.^10^^,^^11^ Additionally, citizen-reported data on the presence of *I. ricinus* indicate a geographical expansion of these ticks towards northern areas in Sweden.^12^ This change in the distribution of vector populations can result in increased exposure to infected ticks in previously non-endemic areas.

Although the first reports of *Borrelia* infections in the United States date back to the 1980s, cases with LB symptomatology had been recorded in Europe as early as the late 19th century.^3^^,^^13^^,^^14^ Since then, most LB cases have been documented in the Northern Hemisphere, with differences between North America and Europe.^1^ While *Borrelia afzelii* and *Borrelia garinii* are the two most common bacteria found in European infections, *Borrelia burgdorferi* sensu stricto is responsible for most infections in North America.^2^ Moreover, the hard ticks responsible for *Borrelia* transmission differ by region: *I. scapularis* and *I. pacificus* are more common in North America, and *I. ricinus* and *I. persulcatus* are the dominant species in European countries.^1^ These differences might explain the distribution of the clinical presentations between continents, with systemic symptoms such as Lyme arthritis and neuroborreliosis being more frequent in Europe.^1^

Endemic circulation of LB in Germany has been reported at least for the last two decades.^15^^,^^16^ According to outpatient claims from health insurance data, more than 128,000 people were diagnosed with LB in 2019 across Germany.^17^ Further estimates from health insurance indicate that the economic cost of LB exceeds 23 million euros each year in treatment of symptoms due to both short and long term *Borrelia* infections.^18^ As a result, LB is recognised as a disease of considerable public health importance. The implementation of national case notification has progressed from two Federal States in 2001 to nine by 2022, reflecting the autonomy each Federal State has in managing its reporting system.^16^

Disentangling the spatiotemporal dynamics of LB and quantifying the influence of environmental and climatic factors is crucial for prevention and informed public health decision-making. Although notification systems may not fully reflect the true epidemiological landscape of LB transmission due to variations in healthcare-seeking behaviour, diagnostic practices, and reporting policies, they offer a structured framework for disease surveillance. Integrating disease surveillance data with meteorological and environmental indicators in predictive models has been instrumental in quantifying the role of multiple risk factors across a wide range of climate-sensitive infectious diseases and in forecasting the likelihood of outbreaks in advance.^19^, ^20^, ^21^ This study aims to quantify the association between environmental and climatic processes and the risk of LB in Germany, while also leveraging these associations to provide insights into areas without mandatory LB notification. Additionally, this study seeks to assess long-term trends in predicted risk given seven decades of climatic data.

## Methods

### Health and population data

We downloaded weekly counts of confirmed LB cases notified to the Robert Koch Institute (RKI) and publicly available on the online platform SurvStat@RKI.^22^ Although surveillance records began in 2001, we considered the period between January 2009 and December 2022, as the latest and most widely accepted case definition was adopted in 2009.^23^ Since then, health professionals diagnose a patient with LB if they present with the characteristic Lyme rash or with symptoms compatible with any of the disseminated forms, along with a confirmatory laboratory test.^24^ By December 2022, national notification of LB had only been implemented in nine of the 16 Federal States. Weekly cases were extracted for 208 districts following the Nomenclature of Territorial Units for Statistics (NUTS3) using the binary male-female sex classification. We excluded cases notified in Saarland to avoid bias, as there were many unexplained records among children aged zero (Supplementary Fig. S2b). We grouped case counts by month according to the date of start of each week. Registries with unknown age or sex were discarded to avoid demographic modelling bias, and the “diverse” category, corresponding to intersex people, was excluded due to the low number of records and its implementation in 2018 (N = 30).

Annual population projections for each NUTS3 district were available from Germany’s Federal Statistics Office and published on the platform Genesis v4.4.2 (Table ID: 12411-0018).^25^ For time series continuity purposes, we adapted the NUTS nomenclature to v2021.^26^

### Climate and environmental data

We extracted climate variables from the E-OBS v27.0e dataset developed within the EU-FP6 project UERRA and the Copernicus Climate Change Service.^27^ We selected a subset of the climate variables based on their relevance in the LB system (Supplementary Table S1), namely daily mean temperature (°C), maximum temperature (°C), minimum temperature (°C), relative humidity (%), and accumulated precipitation (metres) at a 0.1° regular grid resolution (roughly 7 × 11 km in Germany), from January 2008 to December 2022.^27^ For each location, we calculated monthly averages of maximum, minimum, and mean temperatures, and relative humidity, along with monthly total precipitation per grid cell. We then aggregated these data into averages for each NUTS3 district using shapefiles from the European Environment Agency, considering the proportion of each grid cell covered by the corresponding polygon.^28^ In addition to precipitation, we included the Standardised Precipitation Evapotranspiration Index (SPEI), an indicator of the accumulated dry or wet conditions in the previous three (SPEI-3) and six (SPEI-6) months.^29^

We downloaded annual land cover classes between 2009 and 2020, with a 30 m resolution.^30^ Annual land cover classes were produced using a machine learning algorithm that combined CORINE, LUCAS, and GLAD Landsat products.^30^ We assumed land cover stratification remained constant between 2020 and 2022. We computed the percentage coverage of each land cover class per district, with classes below 5% coverage being set to zero to avoid outliers. For the same reason, we excluded land cover classes present in fewer than ten districts per year. Additionally, we combined coniferous, broad-leaf, and mixed forest types into a single category, labelled ‘forest coverage’. A total of nine land cover classes were considered, including agriculture with significant natural vegetation, complex cultivation patterns, forest coverage, green urban areas, industrial or commercial units, non-irrigated arable land, pastures, road and rail networks and associated land, sport and leisure facilities, urban fabric, and water bodies (Supplementary Table S2). Furthermore, we incorporated a red deer (*Cervus elaphus*) habitat suitability (DHS) index created for Europe in 2014 at a 1 km resolution, which we summarised as the mean per district.^31^ Due to the lack of a temporal structure in this index, we assumed it remained constant throughout the study period (Supplementary Fig. S1).

### Statistics

#### Modelling framework

We built a set of hierarchical mixed models within a Bayesian framework to estimate the risk of LB case reports to the national notification system, hereafter referred to as LB risk or relative risk. We assumed infections happened independently at random, and their reporting probability to be uniform. Thus, we modelled monthly case counts between January 2009 and December 2022, in each of the districts, as following a negative binomial distribution. We included spatial and temporal random effects to account for unmeasured variability in the data. First, we used a cyclic second-order random walk (*c*RW2) model for months, which is useful when there is a lack of information on seasonal drivers of disease, such as movement of tick hosts, or holiday seasons that increase the amount of people performing outdoor activities. Likewise, we accounted for unmeasured spatial processes at a district level using a modified Besag-York-Mollie model (*m*BYM), which jointly models spatially structured processes, such as sharing similar socio-economic characteristics between neighbouring areas, as well as unstructured variation in spatial dynamics, such as differences in public health systems. As the geographical coverage of the mandatory notification system changed between years, we replicated the spatial effects yearly to include unmeasured interannual variability. We fitted the models and extracted the marginal posterior distribution of coefficients using the Integrated Nested Laplace Approximation (INLA).^32^ Further specifications on the modelling framework and the random effect definitions can be found in the Supplementary materials.

To ensure that our model structure aligned with established epidemiological knowledge of LB transmission, we first reviewed the literature on environmental and climatic drivers of tick dynamics and human exposure (Supplementary Tables S1 and S2).

We then applied statistical model selection criteria to refine our multivariable model, ensuring that the final structure captured known ecological drivers with optimal predictive performance. Following Gibb et al., 2023, the baseline model comprised temporal and spatial random effects exclusively.^21^ Next, we assembled a set of univariable models incorporating climatic and environmental variables, based on previously documented drivers of disease in the literature, to quantify their associations with notified cases. We tested both linear and non-linear relationships while exploring potential time lags of up to six months. We used a RW2 model to include nonlinear associations. We performed model selection using a range of goodness-of-fit statistics: the Watanabe-Akaike Information Criteria (WAIC), the Deviance Information Criterion (DIC), the log score of the Conditional Predictive Ordinates (CPO), and the Mean Absolute Error (MAE). We selected univariable models with better performance in at least three of the four statistics and combined them into a multivariable candidate model.

To avoid collinearity, we assessed both linear and nonlinear correlations between the candidate variables prior to including them in the multivariable model. We computed correlations using adaptive local linear correlation computation, which partitions the data into subsets to compute multiple linear correlations.^33^ If two or more variables were highly correlated, we then retained those with the largest significant effect sizes. To evaluate the relevance of each variable in the final model, we conducted tests by systematically removing each variable one at a time. If the exclusion of a variable strictly improved the model fit, it was excluded from the final model.

As the model complexity increased with the inclusion of explanatory variables, we anticipated changes in the posterior contribution of the random effects relative to the baseline model. We examined the mean and 95% credible intervals of the posterior distribution of three parameters in each model. First, we observed the precision parameter (τ), defined as the inverse of the variance. A higher value of τ indicated a smoother fit of the random effects function and, hence, a greater certainty in the estimates across spatial or temporal units.^34^ Second, we extracted the mixing parameter (ϕ), which balances the relative contributions of the spatially structured and unstructured components of the model. Values of ϕ closer to one suggested a larger contribution from the structured component of the *m*BYM model, while values closer to zero indicated a greater contribution from the unstructured component.^35^ Finally, we assessed the overall effect of the functions by computing the MAE between all the district- or month-specific posterior effect sizes. Lower MAE values implied a smaller contribution from the random effects.^21^

In addition, the correlation between climate and risk may vary across different demographic and environmental settings. First, we stratified the effects of every climate variable in the final model by sex groups and assessed the differences in the posterior effect sizes. Similarly, we stratified the effects of climate between districts classified as suitable or unsuitable for red deer habitat based on the DHS. We determined district-level suitability when the DHS coverage exceeded the median of the country-level DHS coverage distribution (Supplementary Fig. S1).

#### Imputation ability

Understanding the influence of climatic and environmental processes on LB risk, offers the opportunity to predict disease dynamics in regions not included in the sample but facing similar conditions. We evaluated the imputation ability of our chosen model by conducting a series of k-fold block cross-validation tests.^21^^,^^36^ In this process, we divided the data into 5-fold series splits of district–year combinations. Each split represented 20% of the dataset for testing, with 80% used for training. We sequentially removed one segment at a time until we produced a dataset consisting entirely of predicted values and repeated this procedure 10 times with different random blocks. During each iteration, we extracted 1000 samples from the posterior marginal distribution of the overdispersion and the mean parameters. We used these samples to produce the posterior predictive distribution of the case counts, which we drew from a negative binomial distribution.

We evaluated the final model’s predictive performance against a baseline model that solely incorporated random effects, employing both the Continuous Rank Probability Skill Score (CRPSS) and the Mean Absolute Error (MAE).^37^ We then derived the posterior predictive distribution of cases in Federal States without mandatory LB notification to the national system between 2009 and 2022, and evaluated their interannual trends using a linear regression model between incidence per 100,000 inhabitants and year at the district level.

### Long term climate-related relative risk

We extracted the fitted risk function of maximum temperature from the multivariable model and calculated the monthly average relative risk (RR) between 1951 and 2022. To describe the historic trend, we fitted a linear regression model at a NUTS3 level and extracted their slopes, together with their significance values. We then computed the percentage change in the average RR for the period 2013–2022, relative to the historical reference period of 1951–1970. Additionally, we evaluated changes in the seasonal variation of disease records between these periods at the Federal State level. The historical reference period was chosen based on the earliest available E-OBS data, prior to the significant acceleration in global temperatures since around 1970. However, it is important to note that this period does not represent a pre-industrial baseline.

### Ethics

Ethics approval was not necessary as data were anonymised and provided as counts by the Robert Koch Institute via the online platform Survstat@RKI.

### Role of funders

The funders of the study had no role in study design, data collection, data analysis, data interpretation, writing of the report, or decision to submit. The corresponding authors had full access to all the data and the final responsibility to submit for publication.

## Results

**Fig. 2:**
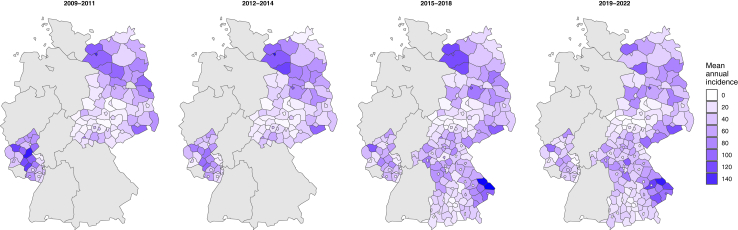
**Mean annual LB incidence per 100,000 inhabitants calculated from mandatory notification data divided in four time periods (2009–2011, 2012–2014, 2015–2018, 2019–2022).** Although LB surveillance dates to 2001, the most recent and widely implemented case definition was adopted in 2009. In addition, LB mandatory notification started in different years for each Federal State.

By July 2023, a total of 123,444 LB cases were notified to the RKI between 2009 and 2022, encompassing 208 districts currently under surveillance (Supplementary Fig. S2a). LB cases were documented across all states, reflecting the endemic nature of the disease in Germany. Cases exhibited a pronounced seasonal pattern throughout the study period, with the highest number of reports occurring between May and December (Supplementary Fig. S3). The highest mean annual incidence was found in districts located in Brandenburg (60 cases per 100,000 inhabitants), Mecklenburg-Vorpommern (52 cases per 100,000 inhabitants), and Saxony (41 cases per 100,000 inhabitants), which are traditionally considered predominantly rural (Fig. 2 and Supplementary Table S3).

After sequentially introducing the explanatory variables, we identified nonlinear associations between LB cases and maximum temperature lagged by two to four months, relative humidity lagged by six months, and SPEI-3 lagged by 1 month (Supplementary Table S4). Due to the minimal impact of total monthly precipitation on WAIC, DIC, and CPO, we retained only SPEI-3, as it already incorporated precipitation.^29^ Environmental models indicated that linear functions had a better fitting performance than the nonlinear formulations. From the different land cover classes, we selected annual forest coverage, urban fabric, industrial or commercial units, road and rail networks associated land, and green urban areas (Supplementary Table S5). Given the strong correlation between the land cover classes ‘urban fabric’ and ‘industrial or commercial units’, we combined their coverage into a single land cover class named ‘urban and industrial fabric’ (Supplementary Fig. S4). The final candidate model included the non-linear formulations of the climate variables and the selected land cover classes, which were retained based on the overall association with LB counts (Table 1).

**Table 1:** Goodness-of-fit statistics in the baseline and the multivariable model.

Model	log(η_t,i_)	DIC	WAIC	CPO
Baseline				
	$\alpha +v_{i\left(a\left(t\right)\right)}+u_{i\left(a\left(t\right)\right)}{+\delta }_{m\left(t\right)}$	99,403	99,531	1.92
Multivariable				
	$\alpha +v_{i\left(a\left(t\right)\right)}+u_{i\left(a\left(t\right)\right)}{+\delta }_{m\left(t\right)}+f\left({TX}_{lag\;2-4}\right)+f\left({RH}_{lag\;6}\right)+f\left({SPEI3}_{lag\;1}\right)+\sum \beta_{k\;a\left(t\right)}{env}_{k}$	98,622	98,760	1.90

Monthly counts of LB were modelled as arising from a negative binomial distribution with mean μ and overdispersion parameter ϕ. The log of the mean risk μ was estimated by incorporating the log of the annual population size per 100,000 inhabitants as an offset, along with a linear predictor, log(η_t,i_), comprising: an intercept (α); spatially unstructured ($v_{i}$) and structured ($u_{i}$) random effects replicated annually (a(t) = 1, …,14) ($v_{i\left(a\left(t\right)\right)}+u_{i\left(a\left(t\right)\right)}$); monthly random effects to capture seasonality (m(t) = 1, …,12) (δ_m(t)_); second-order random walk functions of maximum temperature (*TX*) averaged between lags two and four, relative humidity (*RH*) lagged by six months, and the standardised precipitation-evapotranspiration index (*SPEI-3*) lagged by one month; and k = 4 annual coverage of land cover classes (*env*) with coefficients β_k a(t)_, namely forest coverage, urban and industrial fabric, road and rail networks associated land, and green urban areas.

The MAE values showed a 15.6% reduction in the contribution of monthly random effects, decreasing from 0.82 in the baseline model to 0.69 in the final model. The precision parameter for the *c*RW2 model increased slightly from τ_*month*_ = 8.84, 95% CrI 3.62–17.42, in the baseline model to τ_*month*_ = 11.32, 95% CrI 4.4–21.45, in the multivariable model. This suggests that part of the seasonal variability in the cases was statistically captured by the covariates, though substantial residual variation remained unexplained (Supplementary Fig. S5a). While the MAE values for overall spatial effects remained unchanged, the MAE of the structured effects in the *m*BYM model decreased by 9.5%, from 0.61 to 0.55. The lower precision of spatial effects in the baseline model (τ_*district*_ = 0.94, 95% CrI 0.86–1.02), compared to the multivariable model (τ_*district*_ = 1.21, 95% CrI 1.11–1.32), indicated that some of the spatial variability was captured by the explanatory variables (Supplementary Fig. S5b). Additionally, the mixing parameter decreased by 17.2%, from ϕ = 0.66, 95% CrI 0.59–0.72, to ϕ = 0.54, 95% CrI 0.48–0.61, indicating that the explanatory variables captured part of the spatially structured variability in the data (Supplementary Fig. S5c).

**Fig. 3:**
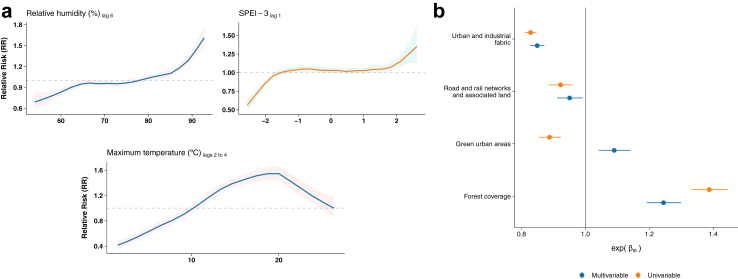
**Relative contribution of variables to LB relative risk.** a. Nonlinear effect sizes of climate variables on relative risk; b. Fixed effects of environmental variables in univariable and multivariable models. SPEI-3: Standardised Precipitation-Evapotranspiration Index accumulated over a three-month period, where extremely negative values indicate exceptionally dry conditions over the past three months, and extremely positive values indicate exceptionally wet conditions.

The association between LB and maximum temperature was positive between 10.5 °C and 26.3 °C, peaking at 20.0 °C. Similarly, we observed an increased mean risk with relative humidity above 78.8%, and positive SPEI-3 values, indicating higher risk after exceptionally wet periods (Fig. 3). Fig. 3 shows the posterior effect of the land cover classes expressed as the exponent of the parameter estimate, exp(β_st_). Positive effects were observed in forested areas (1.24, 95% CrI 1.19–1.3), and green urban areas (1.09, 95% CrI 1.04–1.09). Conversely, there was a negative association with urban and industrial fabric (0.85, 95% CrI 0.83–0.87), and road and rail networks and associated lands showed a null effect in the multivariable model (0.95, 95% CrI 0.92–1). Overall, the effect of the land cover classes decreased in the multivariable model, except for green urban areas, which became positive.

**Fig. 4:**
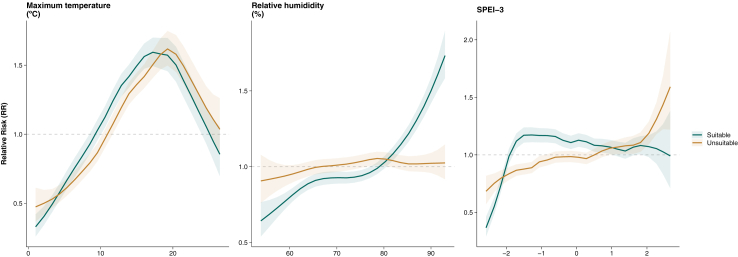
**Effects of climate on LB relative risk stratified by deer habitat suitability.** A district was classified as deer suitable when the district-specific percentage coverage of DHS surpassed the median of the country-wide DHS. Lines represent the mean of the posterior marginal distribution of each climate variable and shaded areas their 95% credible intervals. Each panel shows the effect of each variable leaving the remaining variables constant at their means. The relative risk was plotted using the multiplicative scale, meaning that it is centred at one (dashed grey line).

Adding the classification of districts based on their suitability for deer populations to the final model revealed that districts classified as suitable had 1.37 times higher risk (95% CrI 1.23–1.53) compared to non-suitable districts. The association with relative humidity in suitable districts increased significantly above 80.3%, while it remained relatively stable around one in unsuitable districts (Fig. 4). The effect of SPEI-3 increased steadily, peaking at values between −2 and zero in deer-suitable districts, and then declined. In unsuitable districts, the RR remained close to one for most SPEI-3 values but showed a sharp increase for values above two. The effect of maximum temperature remained consistent across both types, increasing steadily until peaking at 19 °C and decreasing afterwards.

**Fig. 5:**
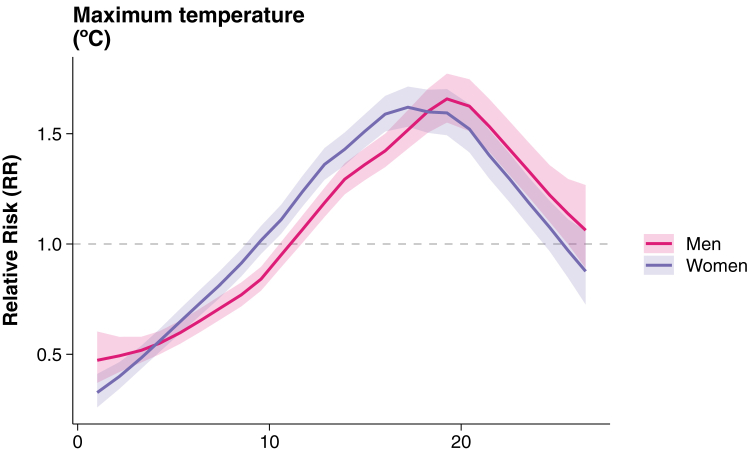
**Effect of maximum temperature on LB relative risk stratified by sex.** Lines represent the mean of the posterior marginal distribution of maximum temperature lagged two-four months, and shaded areas their 95% credible intervals. The relative risk was plotted using the multiplicative scale, meaning that it is centred at one (dashed grey line).

Overall, women experienced a 1.17 (95% CrI 1.16–1.19) times higher risk than men. There was a small difference in the exposure-response association between sex specific LB risk and maximum temperature. At temperatures below 15 °C, women had a slightly higher RR compared to men; however, men showed higher RR above 19 °C. Although credible intervals overlapped through most of the temperature range, there were differences close to the peak, around 19 °C (Fig. 5).

The final model exhibited improved imputation performance compared to the baseline across all iterations, with a median increase in CRPSS of 7.3% (ranging from 7% to 7.75%) and a reduction in MAE by 8.7% (ranging from 7.9% to 9.2%). Observed climatic and environmental conditions in states without reporting indicated that the highest annual rates were in Schleswig–Holstein (45.4 expected cases per 100,000 inhabitants, ranging from 25.3 to 73.2 per 100,000), Hamburg (33.8 per 100,000 inhabitants, ranging from 22 to 56.7 per 100,000), and Lower Saxony (27.9 expected cases per 100,000 inhabitants, ranging from 18.4 to 47.7 per 100,000) (Supplementary Table S6). The linear regression analysis of incidence trends over the years revealed the highest median annual percentage change in Schleswig–Holstein (28.9%, ranging between 10.5% and 41.2% between districts) (Supplementary Fig. S6 and Table S6).

**Fig. 6:**
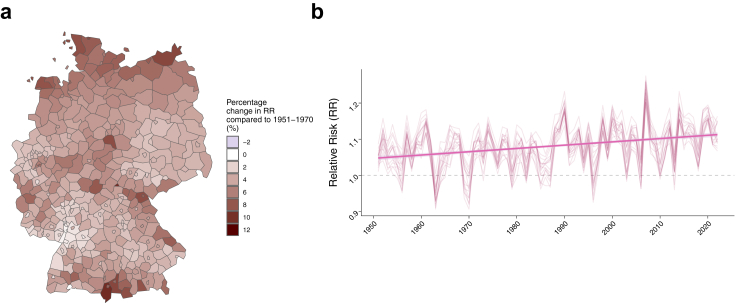
**Historic trend in maximum temperature related LB relative risk.** Using the nonlinear function fitted to maximum temperature in the multivariable model, monthly LB relative risk between 1951 and 2022 was computed for each NUTS3 district. a. Percentage change in the average risk for the period 2013–2022 compared to the reference period 1951–1970. b. Time series of monthly relative risk between 1951 and 2022 per Federal States. The straight line represents the slope of a linear model between relative risk and year (2009–2022).

Compared to the reference period of 1951–1970, the years 2003–2022 have seen increases in maximum temperatures across all districts, ranging from 9.7% to 20.6% (Supplementary Fig. S7). We used the fitted risk function for maximum temperature from the multivariable model to calculate LB RR trends since 1951. We observed geographic variation in the estimated percentage change in RR across the country. Compared to the period 1951–1970, districts in Schleswig–Holstein (6.95%, ranging between 5.77% and 8.63%), Lower Saxony (6.74%, ranging between 5.36% and 8.81%), and Bremen (6.08%, ranging between 5.02% and 7.14%) showed the largest increases (Supplementary Table S7, Fig. 6). Additionally, the average number of months per year with RR above one increased across all Federal States between 3% and 19% (Supplementary Table S7), mirroring the change in temperature (Supplementary Fig. S8).

## Discussion

Tick-borne diseases are a significant concern in the Northern Hemisphere in the context of climate and land use change. In this study, we aimed to quantify the associations between climatic and environmental conditions and Lyme borreliosis (LB) cases reported to the national notification system in Germany. Our research identified conditions positively linked to the risk of LB and leveraged these associations to make predictions in areas without LB records, while also providing insights into historical trends in disease risk.

While most research has focused on the influence of climate and environmental processes on tick population dynamics and infection rates, few studies have successfully identified an association between LB incidence and abiotic factors in Europe. In Sweden, where LB risk follows the distribution of *I. ricinus*, an association was found between the occurrence of Lyme rash and monthly mean summer temperatures as well as summer precipitation.^38^^,^^39^ Similarly, studies using historical LB surveillance records in Hungary and Norway revealed an association between temperature and disease dynamics, with earlier peaks in LB incidence during warmer years.^40^^,^^41^ In Germany, studies using statutory health insurance data identified clusters of LB risk across the country, highlighting high-risk landscapes, such as forested areas and agricultural grasslands.^17^ Although based on notification records, our findings identified similar associations with forested areas, rural districts, and warm, humid conditions, reinforcing the role of landscape and climate in disease dynamics.

The tick life cycle is primarily constrained by the risk of desiccation, with humidity playing a relevant role in survival. Our results suggest an association between higher humidity and increased LB risk. Given that relative humidity above 70% is optimal for *I. ricinus* ticks and that prolonged droughts lead to a decline in tick populations, we hypothesise that the lagged effect of humidity on LB risk in Germany may be linked to tick survival.^6^^,^^8^^,^^42^ However, this relationship may also reflect other ecological and human behavioural factors that were not directly measured in our study, such as a reduction in the frequency of outdoor activities during dry conditions due to the risk of wildfires or landscape changes.^43^

Temperature influences multiple aspects of tick ecology and human exposure patterns. Warmer temperatures have been shown to affect tick development rates and activity levels.^42^ Evidence suggests that temperatures of at least 6 °C are required for ticks to engage in feeding behaviour, although their tolerance to temperature fluctuations allows them to adapt to different thresholds within a few generations.^44^ Higher temperatures may lead to an earlier onset of tick activity in spring, with shorter transition periods between developmental stages.^45^ In parallel, the frequency of outdoor activities undertaken by the German population positively correlates with warmer temperatures, with preferred values between 18 °C and 28 °C.^46^^,^^47^ However, quantifying human exposure to tick bites is difficult due to the variety of transmission settings.^3^^,^^43^^,^^48^ Tick bites can occur during occupational activities, such as farming, or recreational activities like forest walks.^49^ Additionally, people living in peri-urban areas may be exposed while gardening or engaging in other outdoor activities.^49^ In our study, we observed an association between increased maximum temperatures and higher LB risk, which may reflect a combination of the factors discussed in this paragraph.

Although *I. ricinus* ticks can breed in the litter layer to a certain extent, large mammals like deer are preferred hosts for mating.^6^ Consequently, deer presence has been suggested as an indicator of tick abundance.^50^ Using a *C. elaphus* suitability index, we classified districts as suitable or unsuitable habitats based on the species’ niche requirements, which typically include forests, mosaic vegetation, and shrublands.^31^ Overall, LB risk was estimated to be higher in districts classified as suitable for deer populations. However, in districts classified as unsuitable, an increased RR was observed during exceptionally dry conditions. We hypothesise that areas with high biodiversity provide a variety of hosts with differing levels of competence, reducing the probability of tick infection in a phenomenon known as the ‘dilution effect’.^51^ Unsuitable areas for red deer, such as green parks, have less biodiversity, hence increasing the probability of tick infection during exceptionally wet periods. The correlations identified in this study reflect complex interactions between biodiversity, tick distribution, host availability, and human exposure, which warrant further investigation. Moreover, our results should be interpreted with caution, as we assumed habitat suitability remained constant from 2009 to 2022, despite the red deer suitability model being developed for 2014.^31^

Previous research has found that most cases are acquired near the area of residence, indicating that living in or near districts with high risk land classes can help identify transmission hotspots.^16^ Although the influence of urban and industrial areas diminished in the multivariable model, the role of green urban spaces became positive. Urban fabric typically consists of impermeable surfaces such as buildings and roads, while green urban areas are vegetated spaces, often used for recreation or ornamental purposes.^52^ After accounting for the overall negative effect of urban and industrial areas, green urban spaces may concentrate residual LB risk. However, the potential of an urban park to become a LB hotspot depends on factors like size, vegetation type, and its connectivity to other green areas.^53^ Future research should explore the transmission mechanisms that differentiate between suburban margins and enclosed parks, as these are crucial for the strategic planning of urban green spaces, urban expansion, and biodiversity conservation.

Over the past seven decades, Europe has warmed at twice the global average rate.^54^ Current evidence shows ticks expanding to higher altitudes and latitudes, suggesting increasingly suitable conditions for the spread of tick-borne diseases.^7^^,^^10^, ^11^, ^12^ Projections indicate this expansion will likely continue, especially in higher latitudes, with *I. ricinus* spreading further by the end of the century.^9^ Our study aligns with the existing evidence, showing that conditions associated with higher LB risk have become more frequent since 1951. Furthermore, tick seasonal activity is primarily driven by temperature, along with other abiotic factors such as daylight duration.^55^ In this study, we did not observe a trend in the incidence of LB notification between 2009 and 2022, which is consistent with the findings from seroprevalence studies.^56^ This suggests that, although conditions may be becoming more conducive to LB, it does not necessarily result in a higher case burden.^47^^,^^57^ Nonetheless, while increasing temperatures may contribute to changes in tick activity and human exposure patterns, additional factors such as land use change and reporting practices likely play a role in observed trends.

As temperatures continue to rise, there is growing concern over the extended duration of tick questing activity during milder winters.^7^ For instance, a study in Hungary using data from the national surveillance system identified a two-to-three-week earlier peak in warmer years, when assessing the period 1998–2010.^40^ Similarly, the analysis of surveillance records between 1995 and 2019 in Norway revealed that the seasonal peak in LB cases shifted to six weeks earlier.^41^ The 2024 Europe report of the Lancet Countdown indicated that 96% of the NUTS3 districts in Europe had an increase in the climatic suitability for *I. ricinus* activity, when comparing 2013–2022 to 1951–1970.^54^ In addition, projections of nymph questing activity in Germany towards the end of the century suggest a shift towards earlier seasons, with significant heterogeneity across the country.^58^ In line with these studies, we observed a widening of the season compatible with high risk as temperatures rise. We also observed an increasing trend in temperatures positively associated with a higher risk since the 1950s, particularly in northern and southern districts.

Serological studies have indicated that men have twice the odds of having antibodies against *Borrelia* compared to women, which contrasts with our findings based on notification data and those derived from health insurance records.^16^^,^^17^ This suggests differences in exposure or immune response that may not be reflected in notification data.^47^^,^^59^ It has been suggested that women may be more likely to report early symptoms, such as skin lesions, while men are more prone to present with disseminated forms of LB, possibly due to differences in healthcare-seeking behaviour.^60^^,^^61^ This gender difference in reporting and clinical presentation may also be influenced by societal norms and varying levels of awareness.

While the coverage of the mandatory notification system limits our understanding of disease patterns across nearly half of the country, we leveraged associations with climate to predict expected trends in areas without records. Our predictions showed an increasing trend in risk in Schleswig–Holstein, aligning with the findings of a study on outpatient claims data that found the highest relative increase in LB incidence between 2010 and 2019 in this state, with roughly 273 cases per 100,000 inhabitants.^62^ We also identified conditions associated with an increased risk in Hamburg, which, despite being a highly urbanised city, has detected the presence of *Borrelia* in *I. ricinus* ticks in public recreational areas.^63^ Lastly, we observed similar trends in Lower Saxony, where serological studies have detected *Borrelia* infection in the population of Hannover, particularly among men older than 40 years old.^47^ These findings indicate the added value of expanding the geographic coverage of the LB mandatory notification system across the country.

Our findings should be interpreted with caution due to several limitations. While notifications reflect real-world disease cases, they are influenced by surveillance coverage, healthcare-seeking behaviour, and regional variations in diagnostic practices. Previous studies have shown that statutory health insurance data capture significantly higher LB case rates than the national notification system, suggesting potential underreporting in certain areas.^17^ Additionally, notification patterns are shaped by diagnostic guidelines, physician awareness and reporting policies, which may differ across Federal States. Furthermore, not all manifestations of LB are of mandatory notification.^64^ In the absence of detailed information on exposure and clinical presentations, our ability to comprehensively assess the spatial and temporal distribution of cases and to distinguish between recent and chronic infections is limited. As a result, while notification-based models can identify broad-scale environmental patterns linked to LB risk, they do not directly measure pathogen transmission dynamics or tick abundance.

Furthermore, our modelling framework, which focuses on ecological associations at the NUTS3 level, does not infer direct causality. The spatial resolution of notification records creates artificial boundaries that do not align with continuous climatic and environmental factors, though the *m*BYM model partially accounts for these effects in the spatial random effects. While our approach identifies associations useful for surveillance, it does not consider individual exposure, vector dynamics, or *Borrelia* prevalence in ticks. Rather than inferring transmission mechanisms, we examined how environmental factors help explain variations in reported LB cases, to assess their relevance for surveillance in different geographic contexts. As an ecological study, our findings are limited to district-level interpretations and do not capture finer-scale dynamics. Although we incorporate epidemiological knowledge to guide variable selection, our approach remains ecological in nature, meaning observed associations do not imply causation.

Our predictions for areas without mandatory notification assumed that associations observed in districts with records would remain consistent, which may not always hold true. Similarly, historical predictions relied on the trends in maximum temperature, without considering changes in land use or other climate variables included in the model. Lastly, the E-OBS products used in our study were developed from meteorological station observations, which have expanded geographically and updated their technology since the 1950s. These changes may introduce inaccuracies when comparing historical data with present-day settings.

In conclusion, our study highlights the importance of climatic and environmental factors in shaping the risk of LB in Germany. By identifying conditions associated with increased case notifications, we provided insights that can support public health surveillance and preparedness efforts. Through predictive modelling, we identified key conditions linked to increased LB risk, offering valuable insights for regions without LB mandatory notification. Our results emphasise the added benefit of expanding surveillance systems and integrating climate data to enhance public health preparedness at both local and national levels.

## Contributors

Martín Lotto Batista, PhD, contributed to the conceptualisation, data curation, formal analysis, investigation, methodology, visualisation, and writing of the original draft, as well as its review and editing. Bruno Carvalho, PhD, participated in validation, writing the original draft, and reviewing and editing. Rory Gibb, PhD, was involved in methodology, formal analysis, validation, and reviewing and editing. Balakrishnan Solaraju-Murali, PhD, contributed to data curation and the review and editing process. Stefan Flasche, PhD, provided supervision and assisted with the review and editing. Stefanie Castell, MD, was responsible for supervision, project administration, and reviewing and editing. Stéphane Ghozzi, PhD, contributed to supervision, methodology, validation, writing the original draft, and reviewing and editing. Rachel Lowe, Prof, led the supervision, conceptualisation, formal analysis, methodology, project administration, and resource allocation, while also contributing to validation, writing the original draft, and reviewing and editing. MLB and SG accessed and verified the underlying data. All authors read and approved the final version of the manuscript.

## Data sharing statement

The original data are publicly available from the online sources listed in the Methods section. The curated data used in this study, along with the R code, are available in the GitLab repository supporting this publication under the GNU AGPLv3 licence: https://earth.bsc.es/gitlab/ghr/lyme-germany-2025, and archived in a permanent repository: https://doi.org/10.5281/zenodo.15195878.

## Declaration of interests

The authors declare no conflicts of interest.
