# Supplementary material for: Modelling the impact of climate and the environment on the spatiotemporal dynamics of Lyme borreliosis in Germany
Source: eBioMedicine. 2025 Apr 28;115:105701. doi: 10.1016/j.ebiom.2025.105701 (PMC12242607; doi:10.1016/j.ebiom.2025.105701)
Supplement: Supplementary Figs. S1–S8 and Tables S1–S7 [file mmc1.pdf]

Supplementary materials: "Modelling the impact of climate and the environment on the spatiotemporal dynamics of Lyme borreliosis in Germany"

Table of content

Model formulation 2
Table S1. Climate variables included in the study. 3
Table S2. Environmental variables 4
Table S3. Mean annual Lyme Borreliosis (LB) incidence rate per 100,000 inhabitants between 2009 and 2022, grouped by Federal State. 5
Table S4. Difference in Watanabe-Akaike Information Criteria (WAIC) in univariable climate models, compared to baseline model. 5
Table S5. Difference in Watanabe-Akaike Information Criteria (WAIC) in univariable environmental models, compared to baseline model. 6
Table S6. Out-of-sample predictions for Federal States without mandatory notification of LB. 6
Table S7. Average change in LB relaive risk (RR) associated with maximum temperature between 1951–1970 and 2003–2022. 7
Figure S1. Deer Habitat Suitability Index (DHS). 7
Figure S2. Frequency of LB in Germany between 2009 and 2022. 8
Figure S3. Seasonal pattern in the log of LB cases per 100,000 inhabitants in Germany from 2009 to 2022, grouped by Federal State. 8
Figure S4. Nonlinear correlation between selected explanatory variables. 9
Figure S5. Comparison of random effect contributions between the baseline and multivariable models 9
Figure S6. Predicted annual change rate in areas without mandatory LB notification. 10
Figure S7. Historical trend in maximum temperature 10
Figure S8. Seasonal change in LB relative risk (RR) between 1951 – 1970 and 2003 – 2022 11
References 11

Model formulation

Assuming Lyme borreliosis (LB) cases happened independently at random, and that their reporting probability was uniform, a set of generalised linear mixed models were built using a Bayesian framework. Monthly LB counts, $Y_{t,i}$ (n=123,444) were modelled as a negative binomial process

$$Y_{t,i}|\mu_{t,i} \sim NegBin(\mu_{t,i}, \kappa)$$

Where $\mu_{t,i}$ is the mean risk of LB in district i and time unit t, and κ is the overdispersion parameter. The mean risk was modelled as the product of

$$log(\mu_{t,i}) = log(p_{a(t,i)}) + log(\eta_{t,i})$$

Where $p_{a(t,i)}$ is the annual population size per district, which was included as an offset in the model, and the general linear predictor $\eta_{t,i}$

$$log(\eta_{t,i}) = \alpha + \delta_{m(t)} + v_{a(i)} + u_{a(i)}$$

With α being the intercept, followed by a set of temporal and spatial random effects. The inclusion of random effects to the linear predictor allows to account for unobserved variability, uncovering latent patterns, either spatial or temporal.[1] To include unmeasured seasonal variation, we used a cyclic second-order random walk function (*c*RW2) for month $\delta_{m(t)}$, where m(t) = 1,…,12 and m(1) corresponds to January. The cyclic formulation sets the periodicity of the seasonal effects via connecting the last month of each calendar year with the first of the next, i.e. December to January. Likewise, to account for unexplained spatial variation across the study area, a modified Besag-York-Mollie model (*m*BYM) was included for the spatial random effects. The *m*BYM model includes the spatially structured processes using a conditional autoregressive model on a neighbourhood matrix, u_i, and the spatially unstructured processes using a IID model, v_i, where i=1,…,208.[2] The latter assumes district-specific effects are independent and identically distributed, drawn from a gaussian distribution with mean 0 and precision τ. As the mandatory national notification system in Germany expanded its geographic coverage over the years, the spatial random effects were replicated independently for each year, making the components of the *m*BYM model $u_{a(i)}$ and $v_{a(i)}$.

Penalised complexity (PC) priors were applied to the hyperparameters of the random effects. These priors help mitigate the risk of overfitting by penalising deviations from the simplest model, i.e. the null contribution of the random effect.[3]

A multivariable model was developed including explanatory variables so that

$$log(\eta_{t,i}) = \alpha + \sum\beta_k x_{k,t,i} + \delta_{m(t)} + \gamma_{a(t)} + v_{a(i)} + u_{a(i)}$$

Where x is a vector of k covariates with regression coefficients β. Environmental and climatic variables were systematically included in univariable models, with the effect of each variable specified either as a fixed linear function, $x_{k,t,i}$, or as a nonlinear function, $f(x_{k,t,i})$. Fixed effects were modelled using a non-informative prior, defined as a gamma distribution with mean 0 and precision 0·01, and nonlinear effects were modelled using a RW2 model with PC priors on the precision of the effects.[1] In addition, the delayed effect of climate variables was assessed using lagged effects of up to six months.

Univariable models were run to select a range of suitable lags and type of associations, either linear or nonlinear. The variable selection was based on the Watanabe-Akaike Information Criteria (WAIC),[4] the Deviance Information Criterion (DIC),[5] the log score of the Conditional Predictive Ordinates (CPO),[6] and the Mean Absolute Error (MAE). The Integrated Nested Laplace Approximation (INLA) was used for computing the posterior distribution of the model parameters.[7]

The original data are publicly available from the online sources listed in the Methods section. The curated data used in this study, along with documented R code, are available in the GitLab repository supporting this publication under the GNU AGPLv3 license (DOI: 10.5281/zenodo.15195878). This work was based on the work done by Gibb et al., 2023, (DOI: 10.5281/zenodo.10159289) and Lotto Batista & Rees et al., 2023 (DOI: 10.5281/zenodo.7865639).

Table S1. Climate variables included in the study.

Climate variable	Rationale
Minimum temperature (ºC)<br>Mean temperature (ºC)<br>Maximum temperature (ºC)	*Ixodes ricinus* ticks are exophilic, with their life cycle being influenced by temperature. Unfed ticks engage in questing behaviour within a specific temperature range, typically between a lower threshold of 10ºC for daily maximum temperature and an upper limit of 35ºC.[8] These ticks are also vulnerable to temperature extremes, with a significant decrease in the number of questing ticks observed following prolonged frost events.[9] Human bites from *I. ricinus* usually occur during recreational or occupational activities in areas where these ticks are prevalent. Based on tick bite records, most exposure occurs during the warmer months when people are more likely to engage in outdoor activities.[10,11]
Relative humidity (%)	Desiccation poses a significant threat to *I. ricinus* ticks. During off-host phases, both unfed and fed ticks thrive best in environments with air humidity levels above 80%.[12] Prolonged exposure to relative humidity below 70% for a month leads to a reduction in the number of questing ticks, with levels below 40% being potentially fatal.[12]
Accumulated precipitation (m)	The influence of precipitation on *I. ricinus* ticks is less evident compared to temperature and humidity. Summer rainfalls have been identified as potential drivers of changes in *I. ricinus* habitat suitability in Europe.[13] Extended rainfall periods can lead to increased humidity and, hence, survival.[14] However, precipitation does not directly reflect water availability and typically has a low correlation with relative humidity on a continental scale.[15] Nevertheless, precipitation may impact the frequency of outdoor activities, thereby influencing the risk of exposure to tick bites.
Standardised Precipitation Evapotranspiration Index (SPEI)	*I. ricinus* ticks are highly susceptible to desiccation, as mentioned above. The SPEI indicates whether the accumulated conditions over a period were exceptionally dry or wet.[16] In exceptionally dry conditions, together with high temperatures, tick activity tends to decrease, returning quickly to normal values after rainfall occurs.[8]

Table S2. Environmental variables

Environmental variable	Rationale
Forest	Forests are complex biomes that provide the essential elements to support populations of *I.* and their hosts.[17] Moreover, forests offer protection against adverse macroclimatic conditions.[17] From the human perspective, forests are frequently used for both occupational and recreational activities, creating an interface for tick bites, especially along forest edges.[18,19]
Pastures	Pastures are generally considered unsuitable for most tick hosts, limiting tick survival.[20] However, livestock can still be vulnerable to questing ticks, and seasonal deer movements may facilitate tick transport into these biomes.[20] From a human perspective, grasslands, particularly in transitional areas, are often associated with agriculture and farming, where encounters with ticks may occur, thereby increasing the exposure to tick bites.[19]
Non-irrigated arable land<br>Complex cultivation patterns<br>Agriculture with significant natural vegetation	Agricultural areas are not typically associated with increased exposure to tick bites.[21] Crops provide less suitable habitats for ticks compared to forests, as they lack a litter layer and offer less favourable microclimatic conditions.[22] However, agricultural practices and the complexity of the crop, such as mixed-use landscapes, may drive the movement and habitat use of tick hosts.[23] Moreover, frequent human activity along the edges of forest patches transitioning into agricultural areas may also increase occupational exposure to tick bites.[19]
Urban fabric<br>Green urban areas<br>Sport and leisure facilities<br>Road and rail network<br>associated land	At first glance, urban environments may seem unsuitable for *I. ricinus* ticks due to the lack of appropriate vegetation and microclimatic conditions. However, many wildlife species commonly found in urban and peri-urban green spaces can serve as tick hosts and reservoirs for tick-borne pathogens.[19] There have been documented cases of these ticks in urban areas with extensive green spaces, as well as in peri-urban regions with green corridors that facilitate the movement of tick hosts and support tick populations between habitat patches.[14,24]
Deer habitat suitability (DHS)	Although deer are incompetent hosts for *Borrelia*, they are important hosts for adult *I. ricinus* ticks.[14] Consequently, deer presence has been suggested as an indicator of tick abundance.[25] The Deer Habitat Suitability (DHS) index is a composite measure based on the ecological niche requirements of *Cervus elaphus*, incorporating factors such as land cover types, distance to human populations, elevation, and documented deer presence. The DHS index represents the percentage of suitable habitat across Europe at a 20 km resolution.[26]

Table S3. Mean annual Lyme Borreliosis (LB) incidence rate per 100,000 inhabitants between 2009 and 2022, grouped by Federal State. Mean annual incidence is calculated by dividing the number of notified cases per State by the population size projected for that year at the Federal State level.

**Federal State**	**Female (N=67824)**	**Male (N=55620)**	**Overall (N=123444)**
Bavaria	35 (27 – 49)	30 (24 – 43)	33 (26 – 46)
Berlin	25 (19 – 31)	20 (16 – 24)	22 (18 – 27)
Brandenburg	66 (40 – 90)	53 (33 – 70)	60 (37 – 80)
Mecklenburg-Vorpommern	56 (35 – 85)	48 (32 – 68)	52 (34 – 76)
Rhineland-Palatinate	34 (16 – 60)	32 (16 – 60)	33 (16 – 60)
Saxony	46 (26 – 64)	36 (20 – 49)	41 (23 – 57)
Saxony-Anhalt	23 (11 – 37)	19 (10 – 26)	21 (11 – 31)
Thuringia	23 (11 – 32)	20 (8 – 26)	21 (10 – 29)

Table S4. Difference in Watanabe-Akaike Information Criteria (WAIC) in univariable climate models, compared to baseline model. The baseline model included spatial and temporal random effects only, and the univariable climate models included random effects and a climate variable. Largest difference highlighted in yellow. Nonlinear associations were explored using a second order random walk (RW2) model. Pr.: precipitation; RH: relative humidity; Min. T.: minimum temperature; Max. T.: maximum temperature; Mean T.: mean temperature; SPEI-3 and SPEI-6: standardised precipitation-evapotranspiration index accumulated in three and six months.

	**Pr.**	**RH**	**Min. T.**	**Max. T.**	**Mean T.**	**SPEI-3**	**SPEI-6**
**Linear**							
Lag 0	1·41	-14·64	-19·32	-24·60	-29·00	-11·97	-25·21
Lag 1	2·59	0·95	1·68	3·83	6·48	-34·67	-49·78
Lag 2	-20·12	1·33	4·17	4·98	5·31	-41·52	-35·18
Lag 3	-17·76	-32·67	-33·86	-12·81	-21·71	-6·27	-7·45
Lag 4	-29·75	-2·06	-41·08	-23·77	-29·36	-5·45	-0·44
Lag 5	-3·58	2·86	-22·76	-7·40	-11·71	1·07	2·04
Lag 6	-21·64	-100·19	-37·68	4·94	0·04	-3·68	-8·80
**Nonlinear**							
Lag 0	7·33	-70·52	-16·43	-14·12	-60·31	-121·46	-75·98
Lag 1	-1·59	-112·34	-200·16	-67·00	-139·72	-151·92	-94·64
Lag 2	-18·53	-128·41	-18·33	-240·96	-182·10	-42·67	-59·29
Lag 3	-12·30	-135·52	-84·49	-239·42	-212·96	-107·05	-39·62
Lag 4	-39·84	-18·60	-44·37	-117·11	-20·45	-25·87	-37·25
Lag 5	3·84	-28·07	-127·03	-171·83	-151·09	-55·85	-56·43
Lag 6	-14·23	-190·36	-112·85	-147·83	-70·46	-39·94	-108·16
Avg lag 2 – 3*				-316·75			
Avg lag 2 – 4*				-428·42			

* Sensitivity analysis of averages between lags of maximum temperature with the largest difference.

Table S5. Difference in Watanabe-Akaike Information Criteria (WAIC) in univariable environmental models, compared to baseline model. The baseline model included spatial and temporal random effects only, and the univariable environmental models included random effects and a land cover class variable. Largest difference highlighted in yellow. Nonlinear associations were explored using a second order random walk (RW2) model.

	ΔWAIC
**Linear**	
Urban fabric*	-25·18
Industrial or commercial units*	-27·12
Green urban areas	-3·07
Non-irrigated arable land	-0·85
Pastures	-1·89
Complex cultivation patterns	0·39
Agriculture with significant natural vegetation	0·37
Forest coverage	-29·09
Water bodies	-0·80
Sport and leisure facilities	-0·60
Road and rail networks and associated land	-3·95
**Nonlinear**	
Urban fabric	-19·98
Industrial or commercial units	-21·76
Green urban areas	3·30
Non-irrigated arable land	3·24
Pastures	4·43
Complex cultivation patterns	6·26
Agriculture with significant natural vegetation	0·78
Forest coverage	-24·72
Water bodies	7·39
Sport and leisure facilities	5·40
Road and rail networks and associated land	2·44

* The land cover classes ‘Urban fabric’ and ‘Industrial or commercial units’ were later merged into a single category called ‘Urban and industrial fabric,’ resulting in a 30·1 decrease in WAIC compared to the baseline model.

Table S6. Out-of-sample predictions for Federal States without mandatory notification of LB. The mean annual incidence rate (IR), along with the range from 2009 to 2022, was calculated using the median of 1,000 samples from the posterior predictive distribution of case counts. The median annual percentage change in LB risk was determined by fitting a linear regression to the log-transformed predicted annual incidence rate per 100,000 inhabitants over the study period.

Federal State	Predicted mean annual incidence rate IR (range)	Median annual change in LB risk % (range)
Baden-Württemberg	19·2 (15·3 – 24·7)	8·22 (-14·5 – 31·1)
Bremen	19·9 (10·5 – 45)	-4·87 (-18·3 – 8·57)
Hamburg	33·8 (22 – 56·7)	24·4*
Hesse	20·4 (15·5 – 25·8)	11·7 (-15 – 63)
Lower Saxony	27·9 (18·4 – 47·7)	13·8 (-27·6 – 45·4)
North Rhine-Westphalia	23·3 (13·3 – 34·2)	15·5 (-20·3 – 68·4)
Schleswig-Holstein	45·4 (25·3 – 73·2)	28·9 (10·5 – 41·2)

* Hamburg is a city-state.

Table S7. Average change in LB relaive risk (RR) associated with maximum temperature between 1951–1970 and 2003–2022. The nonlinear climate function fitted to maximum temperature in the final multivariable model was used to estimate the historic annual RR since 1951, averaged by Federal State. Changes in seasonality were determined by counting the number of months per year with an RR above one per district, and the results are reported as the mean percentage change per Federal State, along with the range.

Federal State	Average change in months with RR > 1<br>% (range)	Average change in RR<br>% (range)
Baden-Württemberg	5·04 (0·69 – 7·59)	3·35 (0·9 – 7·36)
Bavaria	3·98 (-0·69 – 9·63)	4·34 (1·65 – 10·21)
Berlin*	4·23	3·7
Brandenburg	3·85 (2·1 – 5·84)	3·93 (2·96 – 5·29)
Bremen	10·32 (9·35 – 11·28)	6·08 (5·02 – 7·14)
Hamburg*	9·56	5·53
Hesse	5·01 (1·4 – 8·03)	3·77 (1·77 – 7·9)
Mecklenburg-Vorpommern	9·72 (6·98 – 14·5)	5·45 (3·88 – 8·52)
Lower Saxony	10·09 (7·41 – 12)	6·74 (5·36 – 8·81)
North Rhine-Westphalia	9·52 (4·38 – 12·93)	3·85 (1·56 – 7·04)
Rhineland-Palatinate	5·01 (1·35 – 7·59)	3·18 (0·59 – 7·22)
Saarland	4·76 (3·45 – 5·63)	2·94 (1·52 – 4·77)
Saxony	4·63 (2·8 – 8·21)	3·7 (2·16 – 6·24)
Saxony-Anhalt	4·68 (1·41 – 7·75)	3·52 (2·7 – 5·27)
Schleswig-Holstein	9·44 (7·41 – 10·94)	6·95 (5·77 – 8·63)
Thuringia	5·13 (0·7 – 8·26)	4·39 (2·16 – 10·02)

* Berlin and Hamburg are city-states.

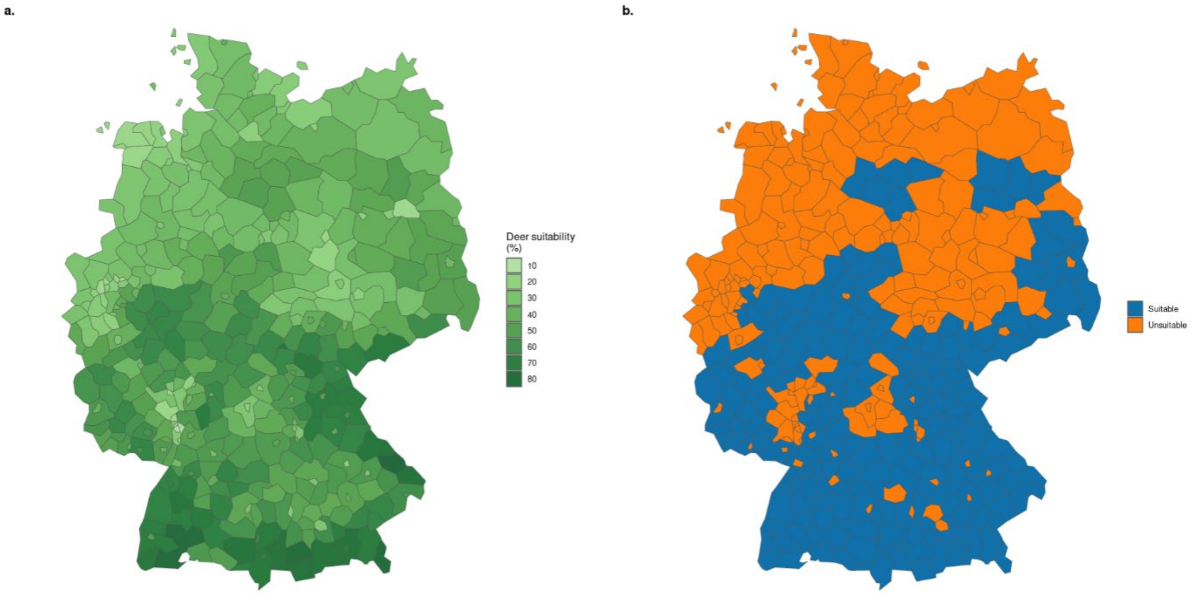

Figure S1. Deer Habitat Suitability Index (DHS). The DHS is a composite measure based on the ecological niche requirements of *Cervus elaphus*, incorporating factors such as land cover types, distance to human populations, elevation, and documented deer presence. The index represents the percentage of suitable habitat at a 20 km resolution.[26] District-level suitability was determined when the DHS coverage exceeded the median value of the country-level DHS coverage distribution.

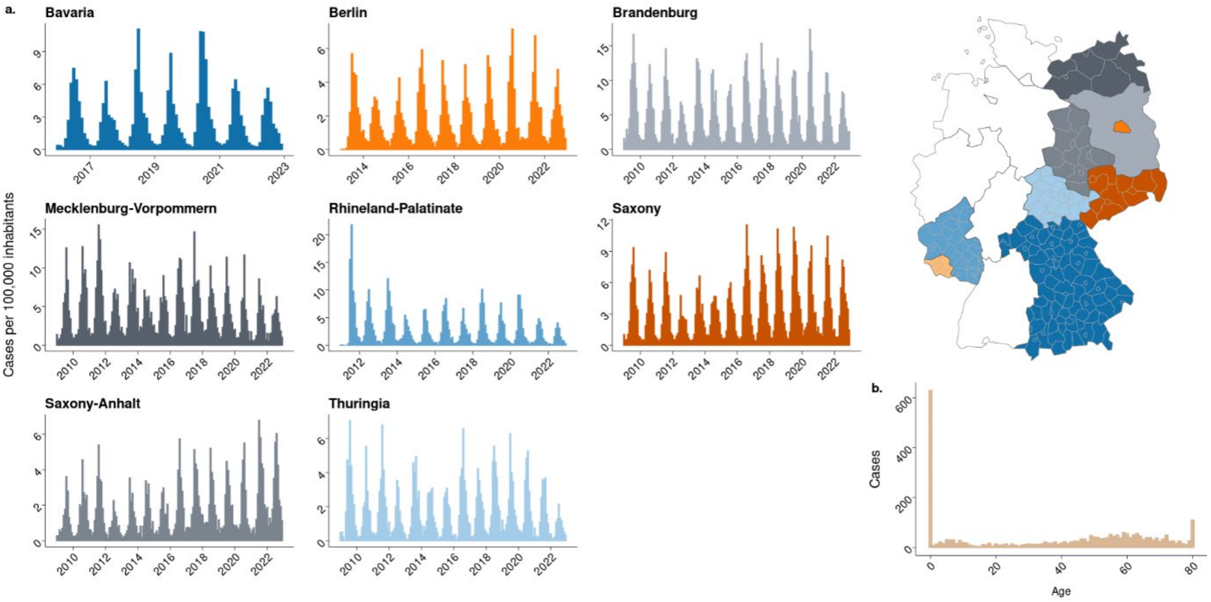

Figure S2. Frequency of LB in Germany between 2009 and 2022. a. Monthly notified LB case counts per 100,000 inhabitants, grouped by Federal State. Due to varying implementation dates of case notification to the national system, the study periods differ across states. b. Age distribution of LB cases in Saarland, highlighting the large number of cases in infants aged zero.

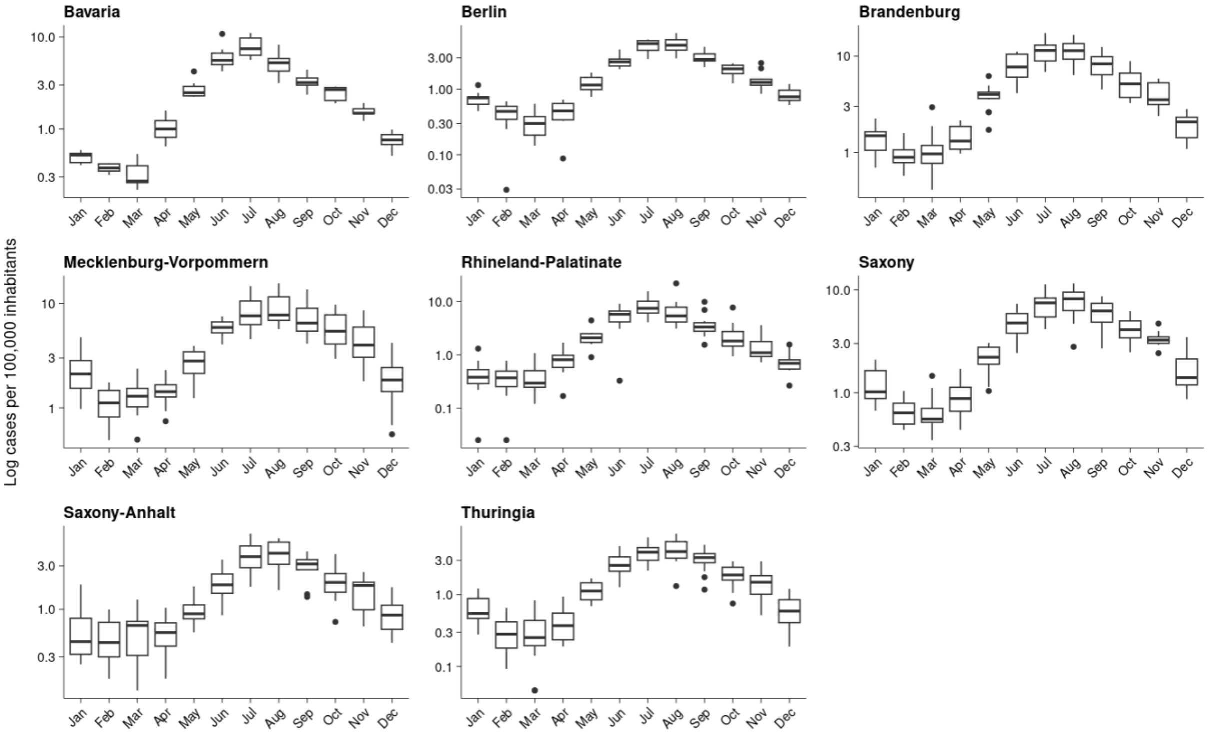

Figure S3. Seasonal pattern in the log of LB cases per 100,000 inhabitants in Germany from 2009 to 2022, grouped by Federal State.

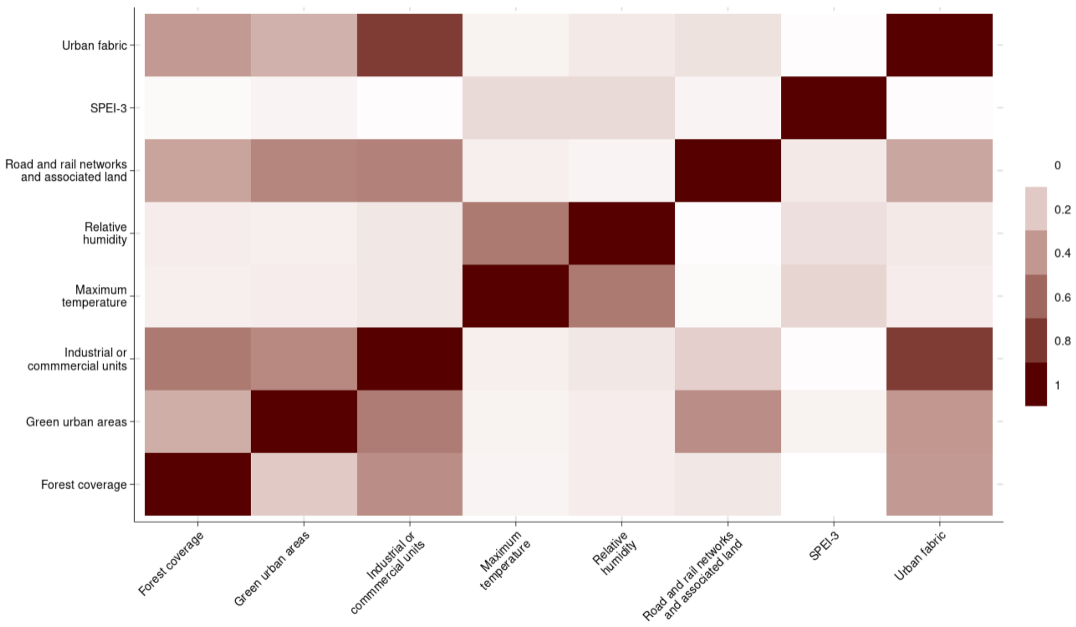

Figure S4. Nonlinear correlation between selected explanatory variables. Correlation analysis used adaptive local linear correlation computation, capable of detecting both linear and nonlinear correlations.

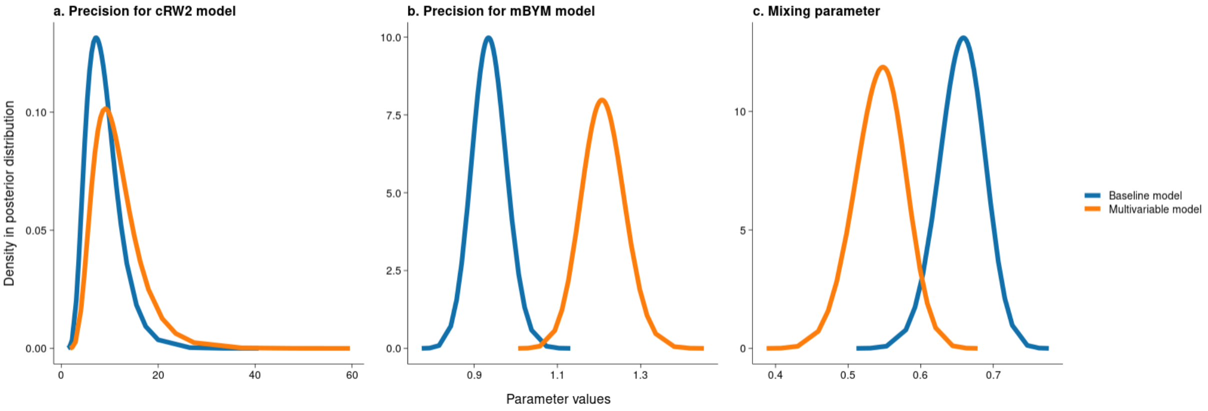

Figure S5. Comparison of random effect contributions between the baseline and multivariable models. Panels (a) and (b) show the precision parameter (τ) for the cyclic second-order random walk (*c*RW2) model and the modified Besag-York-Mollié (*m*BYM) model, respectively. The precision, defined as the inverse of the variance, controls the variability in the random effects. A higher τ value indicates greater certainty in the effect estimates across spatial or temporal units. Panel (c) displays the mixing parameter (ϕ), a coefficient integrated into the *m*BYM model that indicates the relative contribution of the structured and unstructured components to the variance in the spatial model. A mixing parameter of zero implies that the spatial distribution of cases is driven solely by district-specific factors (i.e., accounting for overdispersion), while a value of one suggests that the spatial distribution is fully explained by the neighbourhood structure.

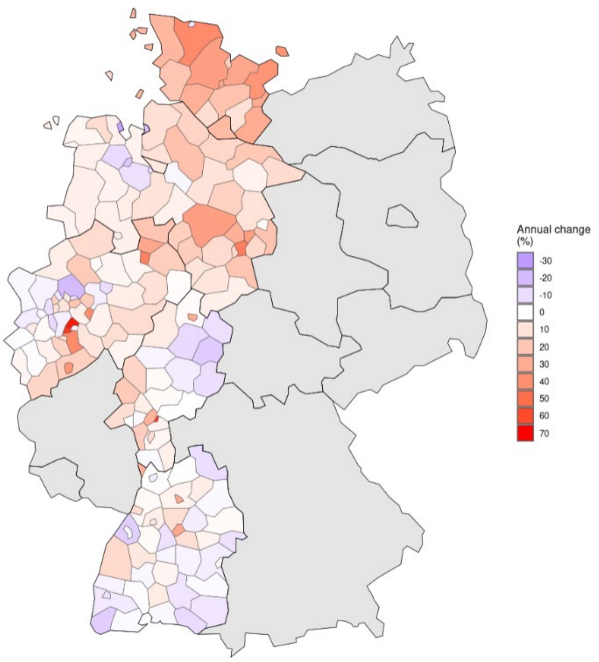

Figure S6. Predicted annual change rate in areas without mandatory LB notification. The annual percentage change in LB risk was determined by fitting a linear regression to the log-transformed predicted annual incidence rate per 100,000 inhabitants for each district over the study period. Case counts were derived from 1000 samples from the model's posterior predictive distribution and divided by the district's population size for that year. To avoid missing values when computing the logarithm, zero counts were transformed into 0·01.

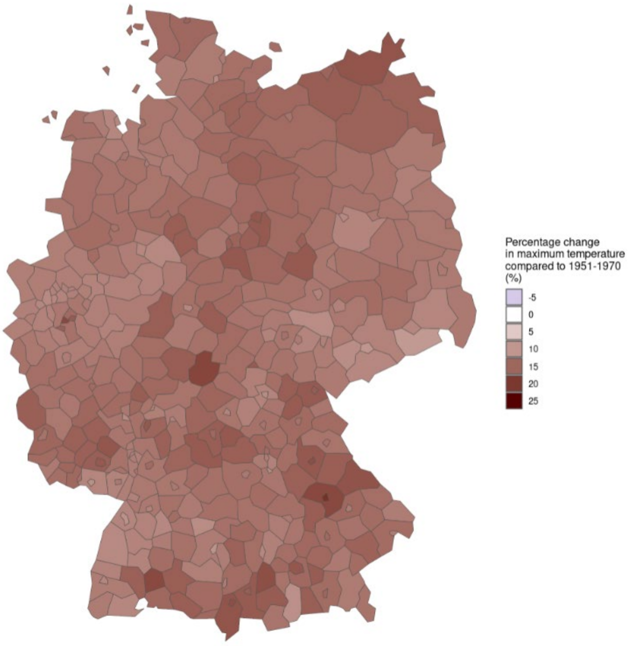

Figure S7. Historical trend in maximum temperature. The average maximum temperature for the period 2013 – 2022 was calculated and compared to the average during the reference period of 1951 – 1970. Results are presented as a percentage change relative to the reference period.

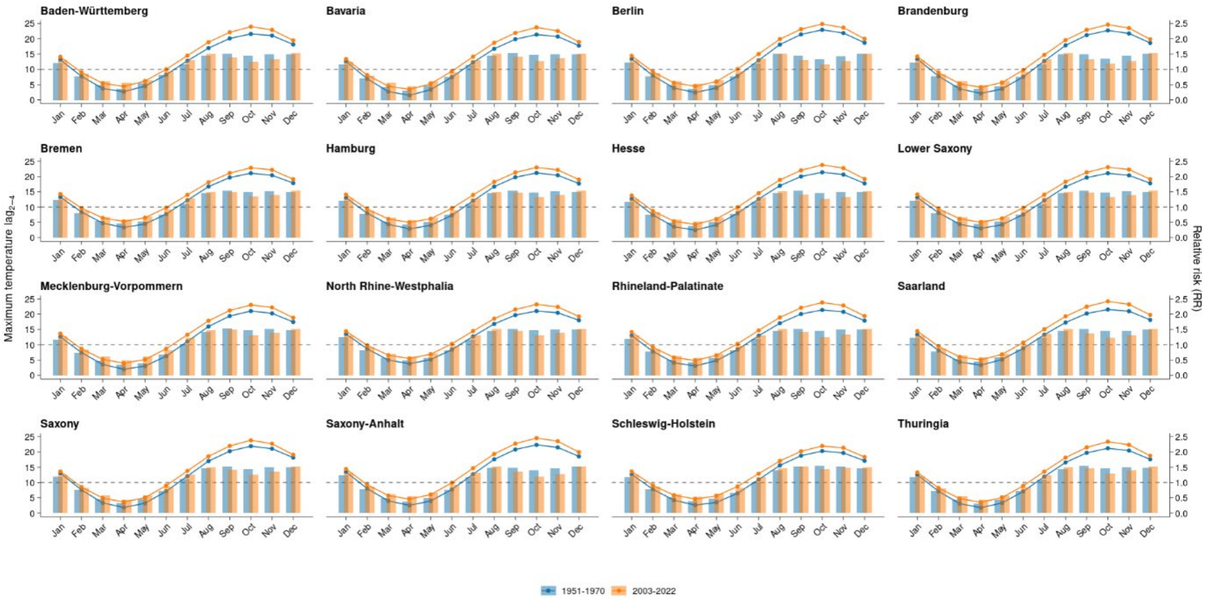

Figure S8. Seasonal change in LB relative risk (RR) between 1951 – 1970 and 2003 – 2022. The nonlinear maximum temperature function in the multivariable model was used to estimate the historic monthly RR since 1951. The change in seasonality was assessed by calculating the average monthly RR for each Federal State over the two periods (bar plot). The change in maximum temperature was determined by averaging the monthly values for each Federal State over the same periods (line plot).

References

1 Blangiardo M, Cameletti M, Baio G, Rue H. Spatial and spatio-temporal models with R-INLA. *Spatial and Spatio-temporal Epidemiology* 2013; **4**: 33–49.

2 Riebler A, Sørbye SH, Simpson D, Rue H. An intuitive Bayesian spatial model for disease mapping that accounts for scaling. 2016; published online Jan 6. DOI:10.48550/arXiv.1601.01180.

3 Simpson D, Rue H, Riebler A, Martins TG, Sørbye SH. Penalising Model Component Complexity: A Principled, Practical Approach to Constructing Priors. *Statistical Science* 2017; **32**: 1–28.

4 Gelman A, Hwang J, Vehtari A. Understanding predictive information criteria for Bayesian models. *Stat Comput* 2014; **24**: 997–1016.

5 Spiegelhalter DJ, Best NG, Carlin BP, Van Der Linde A. Bayesian measures of model complexity and fit. *Journal of the Royal Statistical Society: Series B (Statistical Methodology)* 2002; **64**: 583–639.

6 Pettit LI. The Conditional Predictive Ordinate for the Normal Distribution. *Journal of the Royal Statistical Society: Series B (Methodological)* 1990; **52**: 175–84.

7 Rue H, Martino S, Chopin N. Approximate Bayesian inference for latent Gaussian models by using integrated nested Laplace approximations. *Journal of the Royal Statistical Society: Series B (Statistical Methodology)* 2009; **71**: 319–92.

8 Kahl O, Gray JS. The biology of Ixodes ricinus with emphasis on its ecology. *Ticks and Tick-borne Diseases* 2023; **14**: 102114.

9 Dautel H, Dippel C, Kämmer D, Werkhausen A, Kahl O. Winter activity of *Ixodes ricinus* in a Berlin forest. *International Journal of Medical Microbiology* 2008; **298**: 50–4.

10 Wilhelmsson P, Lindblom P, Fryland L, *et al.* Ixodes ricinus ticks removed from humans in Northern Europe: seasonal pattern of infestation, attachment sites and duration of feeding. *Parasites & Vectors* 2013; **6**: 362.

11 Garcia-Marti I, Zurita-Milla R, Harms MG, Swart A. Using volunteered observations to map human exposure to ticks. *Sci Rep* 2018; **8**: 15435.

12 Grigoryeva LA. Influence of air humidity on the survival rate, lifetime, and development of Ixodes ricinus (L., 1758) and Ixodes persulcatus Schulze, 1930 (Acari: Ixodidae). *saaa* 2022; **27**: 2241–8.

13 Estrada-Peña A, Venzal JM. Changes in Habitat Suitability for the Tick Ixodes ricinus (Acari: Ixodidae) in Europe (1900–1999). *EcoHealth* 2006; **3**: 154–62.

14 Medlock JM, Hansford KM, Bormane A, *et al.* Driving forces for changes in geographical distribution of Ixodes ricinus ticks in Europe. *Parasit Vectors* 2013; **6**: 1.

15 Estrada-Peña A, Estrada-Sánchez A, Estrada-Sánchez D. Methodological caveats in the environmental modelling and projections of climate niche for ticks, with examples for *Ixodes ricinus* (Ixodidae). *Veterinary Parasitology* 2015; **208**: 14–25.

16 Vicente-Serrano SM, Beguería S, López-Moreno JI. A Multiscalar Drought Index Sensitive to Global Warming: The Standardized Precipitation Evapotranspiration Index. *Journal of Climate* 2010; **23**: 1696–718.

17 Gethmann J, Hoffmann B, Kasbohm E, *et al.* Research paper on abiotic factors and their influence on Ixodes ricinus activity-observations over a two-year period at several tick collection sites in Germany. *Parasitol Res* 2020; **119**: 1455–66.

18 Sandifer PA, Sutton-Grier AE, Ward BP. Exploring connections among nature, biodiversity, ecosystem services, and human health and well-being: Opportunities to enhance health and biodiversity conservation. *Ecosystem Services* 2015; **12**: 1–15.

19 Rizzoli A, Silaghi C, Obiegala A, *et al.* Ixodes ricinus and Its Transmitted Pathogens in Urban and Peri-Urban Areas in Europe: New Hazards and Relevance for Public Health. *Frontiers in Public Health* 2014; **2**. https://www.frontiersin.org/articles/10.3389/fpubh.2014.00251 (accessed Feb 22, 2023).

20 van Oort BEH, Hovelsrud GK, Risvoll C, Mohr CW, Jore S. A Mini-Review of Ixodes Ticks Climate Sensitive Infection Dispersion Risk in the Nordic Region. *International Journal of Environmental Research and Public Health* 2020; **17**: 5387.

21 Ehrmann S, Liira J, Gärtner S, *et al.* Environmental drivers of Ixodes ricinus abundance in forest fragments of rural European landscapes. *BMC Ecol* 2017; **17**: 31.

22 Medlock JM, Vaux AGC, Hansford KM, Pietzsch ME, Gillingham EL. Ticks in the ecotone: the impact of agri-environment field margins on the presence and intensity of Ixodes ricinus ticks (Acari: Ixodidae) in farmland in southern England. *Med Vet Entomol* 2020; **34**: 175–83.

23 Panzacchi M, Linnell JDC, Melis C, *et al.* Effect of land-use on small mammal abundance and diversity in a forest–farmland mosaic landscape in south-eastern Norway. *Forest Ecology and Management* 2010; **259**: 1536–45.

24 Heylen D, Lasters R, Adriaensen F, Fonville M, Sprong H, Matthysen E. Ticks and tick-borne diseases in the city: Role of landscape connectivity and green space characteristics in a metropolitan area. *Sci Total Environ* 2019; **670**: 941–9.

25 Jaenson TGT, Eisen L, Comstedt P, *et al.* Risk indicators for the tick Ixodes ricinus and Borrelia burgdorferi sensu lato in Sweden. *Medical and Veterinary Entomology* 2009; **23**: 226–37.

26 Wint W, Morley D, Medlock J, Alexander NS. A First Attempt at Modelling Red Deer (Cervus elaphus) Distributions Over Europe. *Open Health Data* 2014; **2**: e1–e1.
